# HRV-based workplace consultation for managers: a randomized controlled trial on enhancing biopsychosocial awareness and reducing perceived stress

**DOI:** 10.3389/fpubh.2025.1707373

**Published:** 2026-02-03

**Authors:** Elisabeth M. Balint, Christin Braun, Charlotte Mümken, Franziska Kessemeier, Thomas Buckley, Eva Urban, Falko Papenfuss, Harald O. Guendel, Marc N. Jarczok

**Affiliations:** 1 Leadership Personality Center Ulm (LPCU), University of Ulm, Ulm, Germany; 2 Center for Mental Health, Privatklinik Meiringen, University of Bern, Bern, Switzerland; 3 Clinic for Psychosomatic Medicine and Psychotherapy, University Medical Center Ulm, Ulm, Germany; 4 Department of Psychology and Psychotherapy, Witten/Herdecke University, Witten, Germany; 5 Faculty of Medicine and Health, The University of Sydney, Camperdown, NSW, Australia; 6 Robert Bosch GmbH, Gerlingen, Germany

**Keywords:** biofeedback, heart rate variability, managers, primary prevention, psychosomatic, self-regulation, stress management, workplace health

## Abstract

**Introduction:**

Effective tools are needed to communicate the biopsychosocial model of health in the workplace, emphasizing the interaction between mind, body, and environment. A 24-h heart rate variability (HRV) spectrogram, combined with a diary, was used to provide individualized feedback on these interactions.

**Methods:**

One hundred managers from a manufacturing and engineering company in southern Germany were randomized into either an intervention group (IG) or a control group (CG). The IG underwent a 24-h ECG measurement, documented daily events, and completed questionnaires. These data informed a 50-min consultation that focused on the connection among emotions, thoughts, recorded situations, and HRV. Questionnaires were repeated after 3 months. Changes in the primary outcome—awareness of biopsychosocial interactions—were analyzed using repeated-measures ANOVA.

**Results:**

Data from 81 participants were included in the final analysis. Participants (94% men, mean ± SD age 48 ± 8.6 years) in the IG reported significantly greater awareness of mind–body interactions (*p* = 0.032, *η*^2^ = 0.057) and were more likely to respond to their body’s needs (*p* = 0.008, *η*^2^ = 0.085). Perceived stress was significantly reduced only in the IG (*p* = 0.017, *η*^2^ = 0.071).

**Conclusion:**

This approach, which integrates HRV visualization with diary-based reflection, proved to be a feasible and effective method for enhancing awareness of biopsychosocial processes, supporting self-regulation, promoting behavioral change, and reducing perceived stress in occupational settings.

## 1 Introduction

Biopsychosocial awareness, defined as the conscious recognition and integration of biological, psychological, and social dimensions of health (1), has been discussed for decades as an essential factor in understanding and managing stress (2), particularly in high-demand occupational settings (3). As a multidimensional construct, it reflects an individual’s capacity to monitor internal states, recognize psychosocial stressors, and interpret physiological cues in context. It represents a basis for psychosomatic health (4, 5). Specifically, emotional awareness has been demonstrated to contribute to improvements in both physical and mental health (6). Given that emotional awareness constitutes only one facet of the complex bio-psycho-social model, a comprehensive understanding of the model as a whole is likely to offer even greater potential for enhancing physical and mental wellbeing.

Despite its relevance, biopsychosocial awareness remains under-addressed in standard occupational health practices, which often emphasize disease detection over preventive engagement. Existing medical checkups largely focus on secondary prevention, identifying health issues after they have developed (7). In contrast, primary prevention requires tools that not only detect early signs of dysregulation but also facilitate reflective insight and motivate behavioral adaptation well before clinical symptoms arise.

Autonomic nervous system (ANS) functioning, as measured, for example, by heart rate variability (HRV), offers a promising physiological indicator in this regard. Because it captures both stress reactivity and recovery capacity, ANS activity can serve as a non-specific yet sensitive marker of biopsychosocial integration, bridging the gap between subjective awareness and objective health status. Heart rate variability (HRV) is a psychophysiological measure that, in addition to integrating mental and somatic health, is easy to obtain, non-invasive, and cost-effective (8). Measures of HRV are already recognized as a valuable tool in occupational medicine and ergonomics, with strong consensus on their applicability across a range of diagnostic and preventive purposes. It can be used, for example, to assess physical and psychological strain during the workday, identify stress-related risk areas in the workplace, evaluate fatigue and recovery patterns, and support individualized recommendations for workplace design and health interventions (9, 10). HRV has previously been suggested for use in employee consultation (11, 12). Cardiac variability measures can provide a window into the functioning of the central-autonomic network, reflecting the body’s capacity to adapt to environmental challenges (13) and to regulate emotions (14). Fast projections of the central autonomic network to end organs occur through the parasympathetic (vagal) and sympathetic branches of the ANS. These two branches are often described as antagonists but are better described as complementary (15). The sympathetic projections to the heart influence end-organ function on a timescale of seconds, whereas vagal projections reach the heart within milliseconds. Whereas “classical” stress theories simplify the acute stress response as an increase in heart rate (HR) and activation of the sympathetic branch of the ANS, these theories ignore the processes apparent within the first 1–2 s of an acute stress response. Here, the tonic vagal inhibition of the intrinsic HR diminishes, allowing HR to increase spontaneously up to the intrinsic frequency of the sinoatrial node, which is around 110/min (16). In everyday life, this (dis)inhibitory control is more nuanced, and using spectral analysis of HRV allows us not only to distinguish parasympathetic from mixed influence but also to visualize the influence of social context on end-organ function (12). Taken together, ANS activity can index the overall functioning and adaptability of the body and mind (17).

Unique to the 24-h HRV method is its capacity to simultaneously capture and visualize the dynamic interplay between the sympathetic and parasympathetic nervous systems over a full diurnal cycle. Unlike single-axis measurements such as salivary cortisol, which involve a significant time lag and only measure the hypothalamic–pituitary–adrenal (HPA) axis output, or selective assessments of isolated physiological systems like electrodermal response or electromyography, continuous HRV offers an unparalleled, non-invasive representation of both strain and recovery capacity. It is therefore comparable to the concept of allostatic load (AL) and can be incorporated into the allostatic load index (ALI) as a potential primary mediator (18). However, AL includes secondary outcomes such as BMI and blood pressure, which lie further along the pathway and already reflect manifest disease when elevated, whereas reduced HRV indicates a potential risk of disease at a much earlier stage (19–21). Moreover, the ALI comprises varying combinations of 38 measurements (18), thereby increasing complexity and hindering comparability. In addition, it constitutes merely a risk parameter that does not capture individual situations or behaviors in terms of their effects on personal stress and recovery. In contrast, the high temporal resolution of 24-h HRV enables the precise time-coupling of physiological states with specific self-documented emotional, cognitive, and situational events, transforming the abstract concept of ANS regulation into a tangible, personalized insight that is central to enhancing biopsychosocial awareness (22).

Therefore, the integrated HRV visualization offers the unique value of objectively presenting ANS dysregulation in relation to specific workplace situations, establishing a strong foundation for a successful, individualized consultation and for enhancing biopsychosocial awareness.

The spectrograph serves as a tool to visualize individual physiological responses in specific contexts, thereby facilitating informed adjustments in attitudes and behaviors. More generally, the primary advantage for clients lies in their ability to reflect on daily routines and their physiological correlates, offering a deeper understanding of cumulative bodily stress, as in the concept of allostatic load (23). Beyond identifying risk behaviors, this method also highlights existing resources, reinforcing positive behaviors rather than focusing solely on negative patterns. This individualized, salutogenic approach surpasses conventional health recommendations by fostering greater credibility, self-awareness, and motivation for sustainable change. As an advanced form of psychosomatic biomonitoring, it integrates both physical and mental health dimensions within public health frameworks, equipping individuals with the knowledge to make informed decisions about their wellbeing and to better balance risks and protective factors (12).

We previously evaluated this 24-h HRV-based biopsychosocial consultation in a pilot study in the occupational setting (11), without a control group, conducted by an occupational health physician. Based on these results, a company’s health service provider (“Betriebskrankenkasse”) expressed interest in expanding this intervention to the management of the company as part of their occupational health management with the aim of improving the managers’ health and indirectly the wellbeing of the employees, as leader wellbeing has been linked to employee wellbeing (24). This study extends the previous pilot study by employing a randomized controlled design. Our goal was to investigate whether HRV-based biopsychosocial consultations increased awareness of interactions among thoughts, feelings, and bodily reactions compared to a control group. Furthermore, we assessed the effect of the intervention on perceived stress.

## 2 Materials and methods

### 2.1 Overview

**Figure 1:**
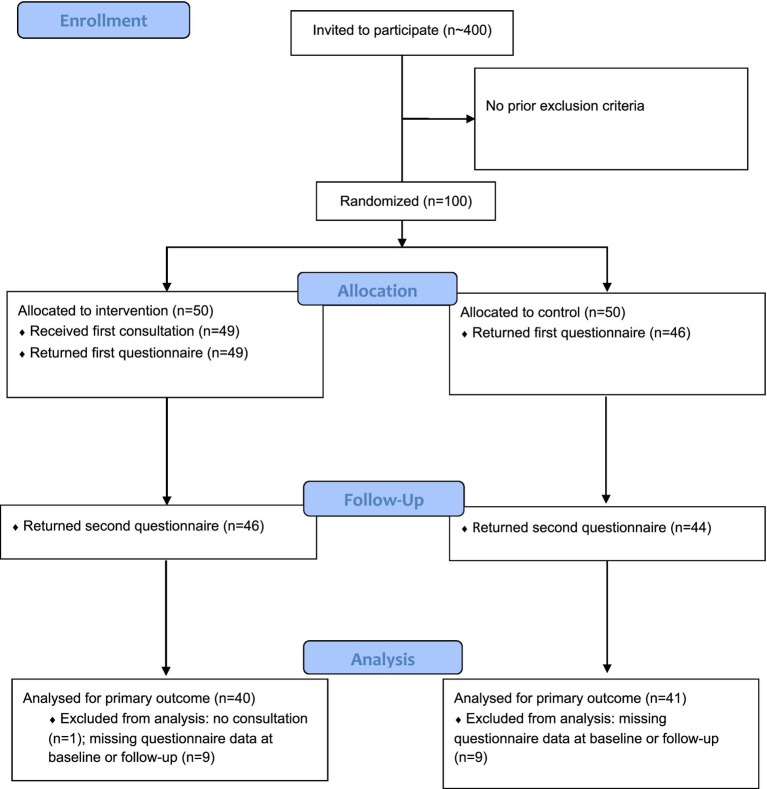
Flowchart of recruitment.

The study was conducted from March to September 2019 at a metalworking company in Southern Germany. It is registered in the German Clinical Trials Register (ID: DRKS00014653) and approved by the local ethics committee (ID 188/18, IRB of Ulm University, Germany). The company was not involved in the study process. The costs of the study were fully covered by a grant from the Karl-Schlecht-Stiftung. Additionally, the company’s health insurance provider supported the study by providing on-site assistance with distributing and collecting study materials. The consultations were conducted in accordance with medical confidentiality. Study participation was offered to all managers (about 400) working at the two local plants of the participating company via email. Exclusion criteria included permanent cardiac arrhythmias (atrial fibrillation) and pacemaker-dependent rhythm. Written informed consent was obtained from managers willing to participate. Randomization of participants into an intervention group (IG) and a control group (CG) was performed by the Institute for Biometry at the University of Ulm, Germany, using a statistical program. Due to the nature of the consultation, blinding of study personnel or participants was not possible. There were no differences between IG and CG regarding sociodemographic variables. For a flowchart of the recruitment process (see Figure 1).

Both groups completed questionnaires at baseline and after 3 months. Participants in the IG received a 24-h ECG recording and protocolled the measurement day. Participants were provided with the equipment by a healthcare professional at their workplace. The counseling physician analyzed the HRV measurement alongside the questionnaire data and the protocol, discussing the results with the participant during a 50-min session. The sessions were conducted either on-site at the company’s premises, in the occupational medicine rooms, or at the LPCU in Ulm, Germany. Afterwards, the HRV consultations were also offered to the CG (data not reported), and the IG received a second HRV measurement and consultation (HRV results are shown in Supplementary Table 1).

### 2.2 Calculation of sample size

An *a priori* power analysis for a repeated-measures ANOVA with a within-between interaction was conducted to determine the required sample size. With an assumed medium effect size (*f* = 0.2), an alpha error probability of 0.05, and a statistical power of 0.95, the analysis indicated that a total sample size of 84 participants (42 per group) would be necessary. This calculation was based on two groups, each measured at two time points, and assumed a correlation of 0.5 among repeated measurements. Given an expected dropout rate of approximately 15%, the number of participants to be recruited was increased accordingly, resulting in a target sample size of 99.

### 2.3 Details of HRV measurement

A single-channel ECG (sampling rate 1,000 Hz) was recorded using a Bittium FarosTM 180 device (Bittium Corp., Oulu, Finland). The selection of this device was based on its suitability for occupational settings: it is small, lightweight, and user-friendly, enabling non-intrusive 24-h measurements without interfering with participants’ daily work routines. The device was either attached using three standard electrodes (Ambu BlueSensorR ECG Electrodes REF R-00-S/25) and a cable set or a textile chest belt with dry electrodes to provide flexibility for participants’ comfort. The entire setup process, including fitting the equipment and providing instructions, was performed efficiently on-site at the company’s premises by the trained staff of the company’s health insurance. This streamlined logistics ensured high participant compliance and minimized disruption to the managers’ workday.

The ECG was imported into CardiscopeTM ANALYTICS Professional Edition (HASIBA medical GmbH, Graz, Austria). Beats were identified as artifacts if the NN interval was shorter than 250 ms or longer than 2,200 ms, or if the maximum NN increase exceeded 132.5%, or if the maximum NN decrease was below 75.5%. The lambda coefficient for detecting premature beats was set to 1.2. A sequence was required to contain at least eight valid beats to be included in the analysis. For analysis, 300-s windows were used, each shifted by 30 s. Within each window, at least 90% of the NN intervals had to be valid. The tachogram was computed using cubic spline interpolation. Frequency-domain analysis was performed using Fast Fourier Transformation (FFT) with a window length of 2048.

ECGs with a high artifact rate were reviewed individually to determine whether sufficient time windows were available for consultation. Since the consultation provides individual situational feedback, roughly half of the total recording time is sufficient for this purpose. In contrast, the criteria for scientific analysis are more rigorous; see the paragraph “HRV analysis.”

### 2.4 Consultation

The consultation was conducted promptly after the measurement, typically within 1 week. It was performed by one of two physicians with training in psychotherapy. The allocation of consultations to one of the two physicians was random. There were no significant differences in the consultees’ baseline variables or in the consultations’ outcome variables between the two physicians. There was a standard procedure for the preparation, implementation, and documentation of the consultation. The German version is available upon request from the authors; a manual for consultations is in preparation. Generally, consultations were prepared by checking the data from the questionnaires, importing the ECG data into CardiscopeTM, and matching the protocol (diary) of the participant with the spectrogram.

The consultation starts with general explanations about HRV and the spectrogram. The main part discusses the spectrogram in terms of interactions among thoughts, emotions, and situational context. This could only be partially prepared, as participants mostly did not note their thoughts and emotions, which had to be explored during the consultation. An example of situations that should have been explored further during the consultation is a period with intensive variability in the spectrograph, followed by a period with low variability, both periods noted as “computer work.” If setting and body position remained the same, but HRV changed, there was often a situational context (a colleague’s email) accompanied by thoughts (how dare he ask me to do that?) and emotions (anger, frustration). The primary aim of the consultation was to visualize this for the participant. Importantly, although stressful or negative moments were explored, they were not labeled “negative.” In contrast, the phase following this stressful event is examined to assess the speed of an individual’s recovery, with successful recovery being highlighted as an indicator of good health. This increased self-efficacy and reduced frustration. Stressors often cannot be avoided. However, recovery can be actively sought afterwards. Therefore, a further aim of the consultation was to detect and strengthen resources and recovery. Recovery moments typically show respiratory sinus arrhythmia within the HF band or an overall increase in variability. If the recorded relaxing activities matched the spectrograph, they were highlighted; if not, they were discussed. A common example was watching TV, which many people label “relaxing,” although HRV often does not align with that perception.

The situations discussed were marked on the spectrograph, and insights were recorded on the report sheet. The latter contained “ressources,” “potentials,” and “goals,” which were noted during the consultation as they were developed together with the client. The participant received the spectrograph and the take-home message.

### 2.5 Psychometric data

#### 2.5.1 Overview

The questionnaire contained standard demographic questions, questions about hours worked weekly, smoking, hours of sports or physical activity per week, knowledge of relaxation methods such as yoga, progressive muscle relaxation, and autogenic training (yes/no), practicing a relaxation method, sleep quality, biopsychosocial awareness, the Irritation Scale (IS) (25, 26), the Perceived Health Questionnaire (PHQ-4) (27, 28), and the Perceived Stress Scale (PSS-4) (29, 30). These questions primarily served as information for the physician preparing the consultation. The questions evaluated in this study are described below.

#### 2.5.2 Primary outcome: biopsychosocial awareness

Biopsychosocial awareness was assessed using the single item “I notice interactions between thoughts, feelings, and bodily reactions in my everyday life” on a Likert scale between “never” (1) and “very often” (10). The selection of a single-item measure was a deliberate choice driven by the importance of feasibility and participant compliance within an organizational context. Measuring biopsychosocial awareness is inherently complex and challenging to operationalize. Although instruments such as the Levels of Emotional Awareness Scale (LEAS) exist, their application was deemed impractical in the current organizational context.

Specifically, the LEAS is considered overly burdensome and not economically feasible in workplace settings, primarily because of the substantial time commitment required from participants, especially leadership personnel, who expressed reluctance to provide extensive written responses. Insights from preliminary studies and pilot testing made it clear that concise and straightforward instruments or questions were necessary to maintain participant compliance. Indeed, using a simpler instrument yielded a retention rate of 81% in the present study. Analogous to the well-established practice of assessing self-rated health using a single-item measure, a single-item approach was deemed suitable for effectively capturing an overall assessment of biopsychosocial awareness. Additionally, at the time this study was conducted, no validated, practical questionnaire such as the “Psychosomatic Competence Inventory” (31) was available.

#### 2.5.3 Secondary outcomes: Self-care

Self-care is represented by the single item “I give my body what it needs,” rated on a Likert scale from “do not agree at all” (1) to “strongly agree” (10).

#### 2.5.4 Secondary outcomes: Perceived stress

Perceived stress was assessed using the Perceived Stress Scale (PSS-4), which comprises four questions asking participants how often they experienced stressful situations in the previous month, rated on a Likert scale from 0 = never to 4 = very often (29, 30). Cronbach’s alpha for this scale is reported as 0.77 (32).

#### 2.5.5 Secondary outcomes: Irritation scale

Irritation was measured using the Irritation Scale, which consists of 8 items assessing cognitive and emotional strain in the work environment (25, 26). Participants rated how frequently they experienced specific thoughts and feelings associated with work-related stress on a Likert scale ranging from 1 = strongly disagree to 7 = strongly agree. The scale comprises two subdimensions: cognitive irritation (e.g., rumination about work-related problems) and emotional irritation (e.g., increased irritability in response to work demands). The total score reflects the overall level of work-related irritation. The scale has demonstrated good internal consistency, with a Cronbach’s alpha of *α* = 0.92 (25).

#### 2.5.6 Comparison to representative data

We compared the study sample with a representative survey of the German general population to evaluate whether the psychological characteristics of the participating managers differed systematically from those of the broader working population. Given the limited number of female participants in our study, the reference sample was restricted to male employees. The comparison was made using weighted norm values from two separate surveys of the German working population. The first survey, conducted in 2021, included 698 male participants and focused on physical and mental wellbeing [Survey No. 31, (33)]. The second survey, conducted in 2019, included 740 male participants and provided additional data on physical and psychological wellbeing (25).

### 2.6 HRV analysis

HRV analysis for scientific purposes (for consultation, see paragraph measurement) was conducted by EB using the CardiscopeTM ANALYTICS professional edition (HASIBA medical GmbH, Graz, Austria). All ECGs had a minimum recording length of 22 h (79,200 s). ECGs were included in statistical analyses only if the sinus rhythm rate was greater than 90%, regardless of the cause (aberrant rhythms or artifacts). Therefore, *N =* 19 24-h ECGs were excluded from this HRV analysis (not from the consultation).

The following parameters were extracted for analysis: RMSSD (the square root of the squared mean of the sum of all differences of successive RR intervals), SDNN (the standard deviation of all RR intervals), SDNN-i (the mean value of the standard deviations of the average RR intervals of all 5-min segments of a measurement), total power-i (average power density in the total band, i.e., between 0.0–0.4 Hz of all 5-min-calculation windows), HFi (Average energy density in the HF (high frequency) band, i.e., between 0.15–0.4 Hz of all 5-min-calculation windows), LFi (average energy density in the LF (low frequency) band, i.e., between 0.04–0.15 Hz of all 5-min-calculation windows), VLFi (average energy density in the VLF (very low frequency) band, i.e., 0–0.04 Hz of all 5-min-calculation windows). Individual HRV values were categorized into age- and sex-specific percentiles using a large, open-access reference table of more than 7,000 employees with 24-h HRV assessments (11).

### 2.7 Statistical methods

For the preparatory analyses, the study sample was compared with a representative German working population survey, restricted to male employees because of the low number of female participants. Differences in demographic and psychological variables were analyzed using independent *t*-tests. To enhance comparability and contextualize HRV measurements within a broader population, individual HRV values were categorized using a large reference dataset (11). This approach facilitates the identification of potential deviations from normative patterns and accounts for naturally occurring variations across different age groups.

The main analyses evaluated the primary and secondary outcomes: biopsychosocial awareness, self-care (after log transformation to fit a normal distribution), and PSS-4 scores. These were compared using a repeated-measures ANOVA. No covariates were included, as IG and CG were very similar. Effect sizes were calculated as *η*^2^, with *η*^2^ = 0.01 representing a small, *η*^2^ = 0.06 a medium, and *η*^2^ = 0.14 a large effect size, respectively. All data management and statistics were conducted using SPSS Statistics for Windows, version 28 (SPSS Inc., Chicago, Ill., USA). A *p*-value smaller than 0.05 (one-sided) was considered statistically significant.

## 3 Results

The included managers were primarily men (*N =* 76, 94%) and living with a partner (*N =* 77, 95%), with an average ±SD age of 48 ± 8.6 years. Most participants worked 40–49 h per week (*N =* 42, 52%). In total, 17 participants (21%) reported more than 49 working hours per week. Eight participants (10%) were active smokers, and 32 (40%) reported <2 h of physical activity per week. The majority of participants (*N =* 67, 83%) reported their sleep as satisfying. Sociodemographic and psychometric data for IG and CG are displayed in Table 1. Dropout analysis (age and sex) revealed no significant differences between the missing data and the analysis sample (data not shown).

**Table 1:** Study population.

Variable	Control group (*N =* 41)		Intervention group (*N =* 40)	
	Mean/N	Standard deviation/%	Mean/N	Standard deviation/%
Age [years]	47.98	9.33	48.70	7.91
Sex [male]	38	93%	38	95%
In partnership	40	98%	37	93%
weekly working hours: > 49 h	8	20%	9	23%
Active Smoking	5	12%	3	8%
Physical activity: > 2 h/week	28	68%	21	53%
Relaxation methods known	30	73%	23	58%
Practicing relaxation methods at least once a week	3	7%	5	13%
Workability [Range 0 to 10]	8.05	1.61	8.07	1.54
Subjective sleep: at least satisfying	36	88%	31	78%
Depressive symptoms (PHQ-2) [Range 0 to 6]	0.93	0.85	1.02	1.23
Anxious symptoms (GAD-2) [Range 0 to 6]	0.83	0.92	0.90	1.01
Cognitive irritation (Irritation Scale) [Range 3 to 21]	10.73	4.44	11.45	4.95
Emotional irritation (Irritation Scale) [Range 5 to 35]	12.95	5.83	11.65	4.57

PHQ, Perceived Health Questionnaire, GAD, Generalized Anxiety Disorder Questionnaire. PHQ-2 and GAD-2 values were calculated as the sum score of the two depression and anxiety questions, respectively.

**Figure 2:**
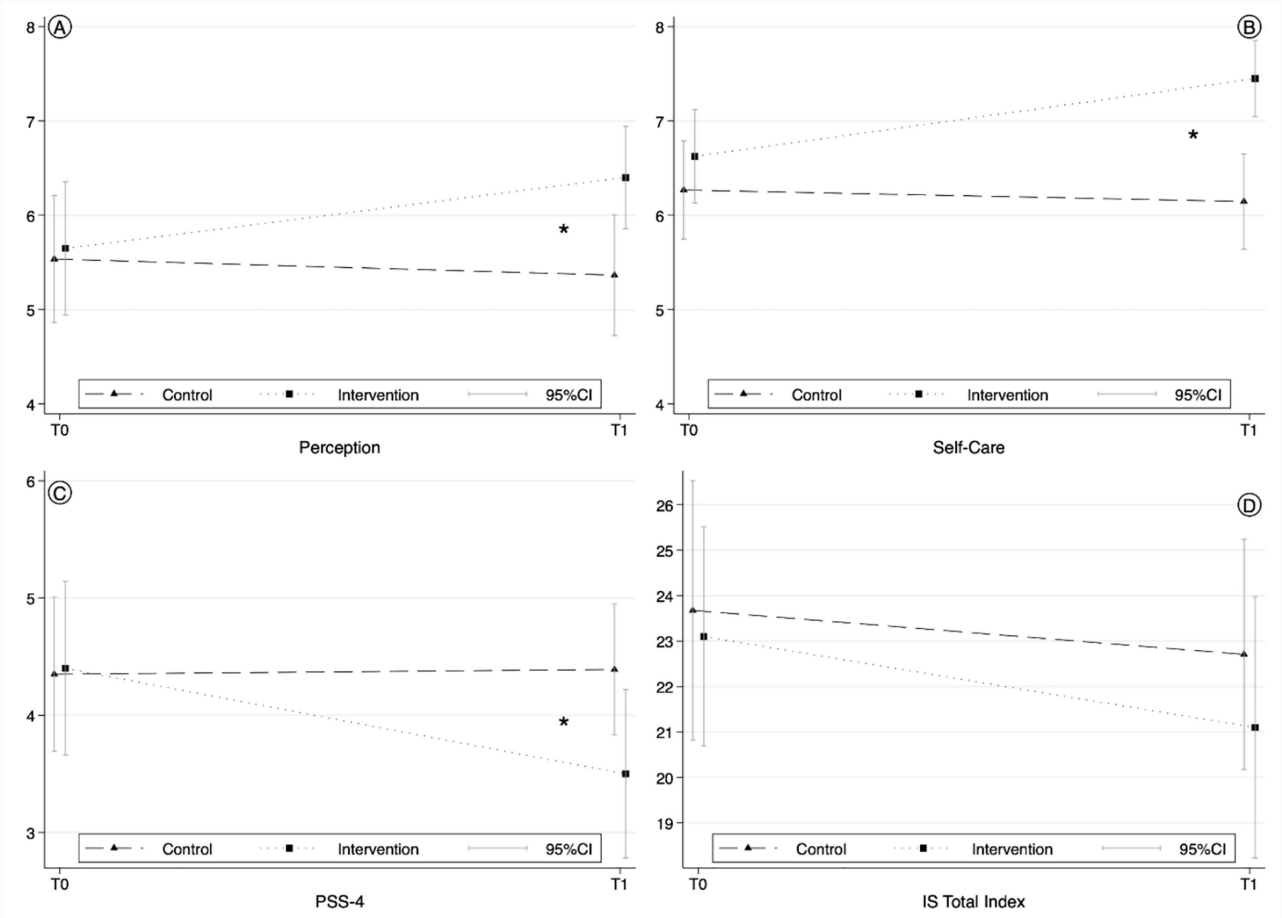
Perception, self-care, stress, and Irritation pre and post intervention for CG and IG. Marginal Means ± Standard Errors from Multilevel Mixed-Effect Linear Regression Models for the **(A)** Perception of biopsychosocial interactions, **(B)** Self-Care, **(C)** Perceived Stress Scale, and **(D)** Irritation Scale at baseline and follow-up for IG versus CG. * indicates *p* < 0.05. **(A)** IG: M = 5.65 ± SD = 2.28 T0, M = 6.40 ± SD = 1.75 T1; CG: M = 5.54 ± SD = 2.20 T0; M = 5.37 ± SD = 2.10 T1; **(B)** IG: M = 6.63 ± SD = 1.60 T0, M = 7.45 ± SD = 1.30 T1; CG: M = 6.27 ± SD = 1.70 T0; M = 6.15 ± SD = 1.63 T1; **(C)** IG: M = 4.40 ± 2.39 T0, M = 3.50 ± 2.32 T1; CG: M = 4.35 ± 2.12 T0, M = 4.43 ± 1.83 T1; **(D)** IG: M = 22.48 ± 7.64 T0, M = 20.83 ± 8.84 T1; CG: M = 23.68 ± 9.20 T0, M = 22.50 ± 8.27 T1.

To address our primary research question, we compared the IG and CG with respect to changes in biopsychosocial awareness, self-care, and perceived stress. Compared to the CG, participants in the IG reported, after the intervention, significantly more often noticing interactions among thoughts, feelings, and bodily reactions in their everyday lives (*F* = 4.77, *p* = 0.032, *η*^2^ = 0.057). They also reported giving their body what it needs significantly more often after the intervention (*F* = 7.34, *p* = 0.008, *η*^2^ = 0.085) (see Figure 2). Both outcomes showed a medium effect size.

Perceived stress was significantly reduced only in the IG, with a medium effect size (*F* = 5.971, *p* = 0.017, *η*^2^ = 0.071). Irritation was not significantly reduced (*F* = 0.205, *p* = 0.652). In general, the CG remained unchanged over the observation period.

Compared to the reference sample, managers in our study were older, scored lower on the Perceived Stress Scale (PSS-4), and reported higher levels of depressive and anxiety symptoms (PHQ), as well as greater irritation (IS) (see Table 2). They were also less likely to recognize their bodies’ needs or respond to them appropriately. Furthermore, they reported perceiving interactions among thoughts, emotions, and bodily sensations less often than participants in the normative sample.

**Table 2:** Comparison of the study population vs. representative sample (men only).

Variable	Study population at baseline		Representative sample^1^		*p*-value (two-sided *T*-Test)
	Mean/N	Standard deviation/%	Mean/N	Standard deviation/%	
Age [years]	48.35	8.53	42.42	12.40	<0.001
Sex [male]		100%	698	100%	
In partnership		95%	644	54%	
Workability [Range 0 to 10]	8.03	1.60	8.29	2.04	0.261
Stress symptoms (PSS-4)	4.45	2.28	5.28	2.66	0.004
Depressive and anxious symptoms (PHQ-4) (sum score)	1.92	1.73	1.07	1.63	<0.001
Depressive symptoms (PHQ-2) (sum score)	1.01	1.05	0.62	0.92	<0.001
Anxious symptoms (GAD-2) (sum score)	0.91	0.97	0.45	0.83	<0.001
Irritation scale sum score	23.85	8.53	17.30^2^	8.90^2^	<0.001
Emotional irritation (IS) sum score	12.61	5.24	10.30^2^	5.50^2^	<0.001
Cognitive irritation (IS) sum score	11.24	4.76	6.90^2^	4.10^2^	<0.001
I can easily perceive what my body needs. Do not agree at all (1), strongly agree (10).	7.41	1.63	8.37	1.71	<0.001
I give my body what it needs. Do not agree at all (1), strongly agree (10).	6.34	1.60	8.14	1.81	<0.001
I notice interactions between thoughts, feelings, and bodily reactions in my everyday life. Never (1) – very often (10).	5.66	2.23	6.14	2.85	0.154

PSS, Perceived Stress Scale; PHQ, Perceived Health Questionnaire; GAD, General Anxiety Disorder Questionnaire; IS, Irritation Scale. ^1^Weighed norm values from a German representative working population sample, men only (*N =* 698), representative survey on physical and mental well-being in the German population No. 31, year of survey 2021 (33). ^2^*N =* 740 (men only), weighed norm values from a German representative working population sample (25), representative survey on physical and mental well-being in the German population No. 28, year of survey 2019.

**Figure 3:**
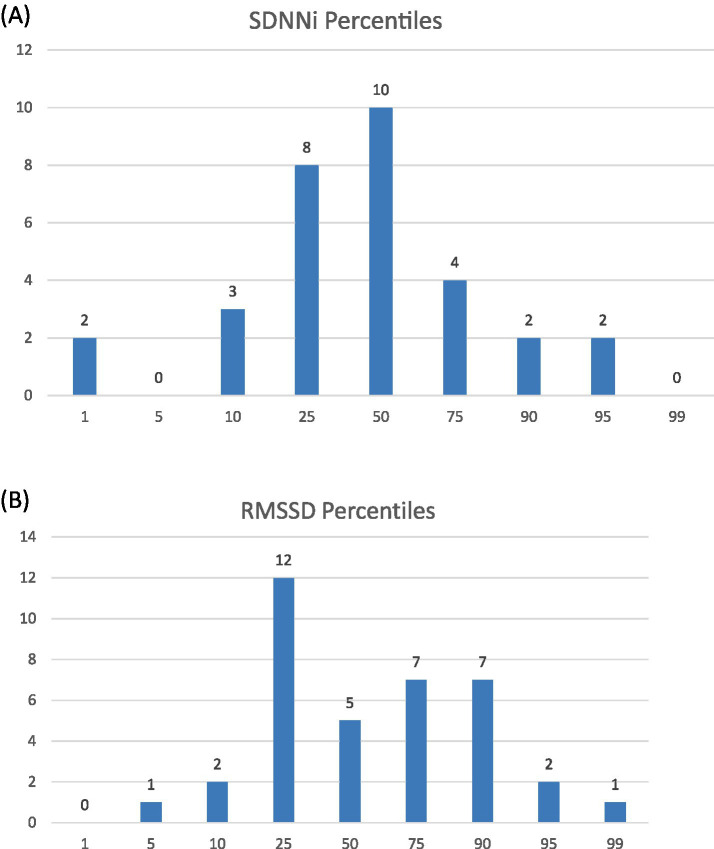
Distribution of SDNNi and RMSSD at baseline, categorized into percentiles based on a reference population. Absolute number of participants with an SDNNi **(A)** and RMSSD **(B)** categorized into sex- and age-specific percentiles based on a reference population (11). Being categorized as 10 represents an SDNNi/RMSSD value between the 10th and 25th percentiles. *N =* 19 ECGs were excluded due to an artifact rate of > 10%.

To assess participants’ autonomic regulation relative to normative values, HRV measurements were categorized into age- and sex-specific percentiles using a large reference dataset comprising more than 7,000 employees with 24-h HRV assessments (11). For details of HRV values in the study population (see Supplementary Table 1). The distribution of SDNN values approximated a normal distribution, whereas the distribution of RMSSD was slightly left-skewed (see Figures 3A,B).

## 4 Discussion

### 4.1 Effects and underlying mechanisms

In this randomized controlled trial, a 24-h HRV-based consultation improved the perception of biopsychosocial interactions in everyday life. Additionally, it enhanced self-care and reduced perceived stress, with medium-sized effects.

To the best of our knowledge, this is the first approach using HRV to visualize biopsychosocial interactions, expanding its application beyond risk assessment and strain determination (9) and sports physiology (34). Beyond mere biofeedback, the goal of the consultation was to develop insights into the complex biopsychosocial interactions that shape health. The strength of this method lies in its high individuality. The situations discussed are specific to the consultee, which is why a single session is often sufficient for the client to transfer this new knowledge to other situations in everyday life.

Other interventions aimed at reducing perceived stress, such as a two-day workshop with two refresher sessions, have been shown to decrease perceived stress reactivity with a small effect size (35), whereas the intervention presented here demonstrated a medium effect size. This difference may be attributable to the individualized counseling approach, in line with personalized medicine, rather than to a standardized workshop where less attention can naturally be paid to individual participants. In a replication (36) of the study (35), conducted by our study group and currently under revision, similar effects and effect sizes were observed as in the original study study (52).

Compared to other interventions using individual biofeedback, a recent systematic review reports medium-to-large effect sizes (37). In these studies, a substantial portion of the training hours was conducted directly by the trainer, supplemented by regular self-practice. Consequently, the effort required from the trainer or counselor is roughly comparable to that of our intervention, while the participant must invest considerably more time. Overall, the effort required by our intervention falls within a typical range for achieving medium effect sizes and is feasible for practical implementation.

Awareness of thoughts, emotions, and somatic reactions is only the first step toward improved health. It is insufficient to know what is beneficial; this knowledge must also be applied. The consultation did not explicitly focus on application, and advice was intentionally avoided. Nevertheless, the clients reported giving their bodies what they needed more often after the intervention. Participants exhibited strong intrinsic motivation to stay healthy and continue performing at a high level, as evidenced during the consultations. This might be a characteristic of the manager population. Nonetheless, it is the cornerstone of any preventive measure to assume that participants have an inherent interest in their own health and will implement measures that seem reasonable and feasible to them.

The relationship between HRV feedback and observed outcomes may be attributable to an improvement in self-efficacy mediated by biofeedback, a mechanism well-established in previous research (38). The high-resolution, personalized visualization of ANS dynamics such as HRV serves as a cognitive anchor, bridging the gap between subjective experiences described in the diary and objective physiological responses reflected in the stress–recovery balance. This personalized insight helps clients manage their physiological state, thereby enhancing their perceived self-efficacy. By highlighting tangible moments of successful recovery and clear triggers for dysregulation, the intervention provides concrete leverage points for effective self-regulation. This increased belief in their ability to successfully execute new, health-promoting behaviors is hypothesized to mediate the increased reported self-care and the subsequent reduction in perceived stress. An association between higher self-efficacy and lower perceived stress has already been demonstrated (39).

In principle, we assume that the intervention also included additional, non-specific therapeutic factors that were not explicitly measured. For instance, interaction with a qualified professional may elicit expectations, and the attention and time provided constitute active components of the intervention, as well-known common factors in psychotherapy (40). From our perspective, these effects are inherently and desirably intertwined with this type of intervention.

We cannot quantify the proportion of the overall effect attributable to these general factors versus the specific contribution of the HRV-based procedure. However, this distinction was not the primary focus of our study. Our main objective was to engage a group that is typically underrepresented in workplace psychosomatic consultations (41) in a psychological consultation to learn about biopsychosocial interactions. Achieving this required more than a simple standard consultation, as managers’ available time is generally very short. To isolate the additional benefit of HRV measurement, a sham intervention would have been necessary, using a randomly generated HRV spectrogram. This was not feasible for the participating companies.

### 4.2 Comparison with other populations and interventions

Compared to a normative sample, the managers in this sample initially showed reduced awareness of their own needs and an increased tendency to ignore bodily demands. This is plausible, as individuals who are promoted are often those who persist in working until a task is completed, even if this requires temporarily neglecting their physical needs. Remarkably, HRV values were only average, despite the executives in this sample reporting higher levels of physical exercise than the general population (42, 43). Given the executives’ above-average healthy lifestyle in terms of physical activity and smoking behavior, above-average HRV values would have been expected. A possible explanation is that increased physical activity, combined with higher psychological stress (elevated PHQ and Irritability Scale scores) and reduced self-care, still has a compensatory effect. This suggests the buffering effect of physical exercise (44, 45).

Finally, perceived stress was also reduced, which is notable since the consultation did not focus on stress reduction but rather on recovery after stress. Although objective stress was not measured, it is important to note that the company where this study was conducted expanded during this time, and many managers had to train external managers in addition to their regular work, suggesting an increase in stress. Two explanations seem possible. First, as the intervention objective helped managers understand their somatic and emotional reactions in interaction with thoughts and circumstances, this might have enhanced their sense of self-efficacy. The reflection of resources and successful recovery may have contributed to a greater sense of control and efficacy in managing stress (46). Second, the consultation might have shifted their focus from stress to recovery, thereby reducing perceived stress (47).

### 4.3 Characterization of the population

The findings of this study should be interpreted in light of several limitations. The study population consisted predominantly of male managers in technical professions. While this may limit the direct generalizability to a broader female workforce, it also represents a distinct strength of our study. The success of this approach in enhancing biopsychosocial awareness and reducing perceived stress was achieved within a population often characterized by a pragmatic, fact-oriented mindset that may be less receptive to psychosomatic concepts. The effectiveness of the HRV-based consultation with this “difficult” target group, which initially showed lower awareness of their own needs than a normative sample, underscores the robustness and power of the method. It suggests that this highly individualized and visual approach, which integrates physiological data with personal experience, has strong potential to succeed with other, potentially more receptive populations and diverse settings as well.

Furthermore, while this RCT focused on managers, the underlying mechanism of enhancing biopsychosocial awareness through personalized physiological biofeedback is theoretically independent of gender or hierarchical status. Our prior work and application experience confirm this: the consultation method has been successfully used by occupational health physicians in other industrial sectors, with populations comprising 35% women and 70% non-managerial employees (11). Moreover, its application has been extended to diverse occupational groups, including police officers (48) and the health sector (49). Thus, we argue that the method’s core functionality for self-regulation and insight generation is broadly generalizable.

Additionally, we did not measure biopsychosocial awareness objectively but relied on self-reports and single, unvalidated items. A limitation of using a single-item measure is that it may fail to adequately capture the nuanced, multidimensional aspects of biopsychosocial awareness. Despite this constraint, the single-item approach proved advantageous due to its high feasibility and significantly improved participant compliance in the organizational setting.

Due to the considerable time required for measurement, assessment, and consultation, it is likely to be reserved for specialized occupational health settings.

While the data suggest that this approach has preventive value, it should be considered only a single, individual intervention and integrated into a broader occupational health management plan (50), including organizational topics and the involvement of occupational health professionals (51).

## 5 Conclusion

The consultation, using a 24-h HRV color spectrogram combined with diary information and a biopsychosocial focus, proved to be a feasible and effective tool for occupational health management, enhancing individuals’ understanding of their unique biopsychosocial interactions. This approach facilitates self-care and reduces perceived stress in a high-demand professional environment. It serves as a gateway for personalized, data-driven preventive strategies and can complement traditional consultations in occupational medicine. Thus, its potential to provide a tangible preventive effect within the framework of corporate health management warrants further exploration in diverse settings to fully assess its broader applicability and benefits.

## Data Availability

The raw data supporting the conclusions of this article will be made available by the authors, upon reasonable request.
