# Supplementary material for: HRV-based workplace consultation for managers: a randomized controlled trial on enhancing biopsychosocial awareness and reducing perceived stress
Source: Front Public Health. 2026 Feb 3;13:1707373. doi: 10.3389/fpubh.2025.1707373 (PMC12909582; doi:10.3389/fpubh.2025.1707373)
Supplement: Supplementary file 1 [file Table_1.docx]

**HRV-based Workplace Consultation for Managers: A randomized controlled trial on enhancing biopsychosocial awareness and reducing perceived stress**

**Supplemental Table 1: HRV-values at baseline and after three months of the IG**

| N=31 | **Median** | **Percentiles** | | **Mean** | **SD** |
| --- | --- | --- | --- | --- | --- |
|  | **50** | **25** | **75** |  |  |
| **T0 rMSSD** | 27.83 | 22.84 | 35.16 | 29.47 | 9.13 |
| **T0 SDNN** | 150.71 | 123.89 | 170.80 | 152.39 | 38.45 |
| **T0 SDNN-i** | 62.29 | 53.26 | 73.04 | 62.24 | 15.00 |
| **T0 HF-i** | 242.79 | 157.47 | 452.01 | 332.74 | 260.81 |
| **T0 LF-i** | 1229.58 | 813.81 | 1920.00 | 1357.73 | 712.14 |
| **T0 VLF-i** | 2063.73 | 1499.07 | 2676.83 | 2256.55 | 1071.92 |
| **T0 TP-i** | 3971.21 | 2733.17 | 5547.00 | 4139.38 | 1933.55 |
|  |  |  |  |  |  |
| **T1 rMSSD** | 27.90 | 23.63 | 31.48 | 29.51 | 9.70 |
| **T1 SDNN** | 144.46 | 128.43 | 174.57 | 151.29 | 36.09 |
| **T1 SDNN-i** | 59.20 | 51.97 | 68.72 | 61.37 | 13.31 |
| **T1 HF-i** | 286.69 | 164.30 | 349.26 | 335.80 | 281.31 |
| **T1 LF-i** | 1273.43 | 811.21 | 1801.57 | 1324.69 | 654.12 |
| **T1 VLF-i** | 1888.91 | 1511.56 | 2541.70 | 2153.47 | 909.98 |
| **T1 TP-i** | 3678.90 | 2844.78 | 4939.34 | 4001.37 | 1715.65 |

Legend: HRV = heart rate variability; SD = standard deviation; RMSSD =square root of the squared mean of the sum of all differences of successive RR intervals; SDNN = standard deviation of all RR intervals; SDNNi = mean value of the standard deviations of the average RR intervals of all 5-min segments of a measurement); TP-i = Average power density in the total band i.e. 0.0-0.4 Hz of all 5-min-calculation windows, HF-i = Average energy density in the HF (high frequency) band i.e. 0.15-0.4 Hz of all 5-min-calculation windows, LF-i = Average energy density in the LF (low frequency) band i.e. between 0.04-0.15 Hz of all 5-min-calculation windows, VLF-I = Average energy density in the VLF ( very low frequency) band i.e. 0-0.04 Hz of all 5-min-calculation windows. For a description of the HRV parameters see (Shaffer & Ginsberg, 2017) and for further information of 24h-analysis see (Jarczok et al., 2019).

Excluded data for this table: N=19 24h-ECGs excluded due to artifact rate > 10%
